# A Systematic Review and Meta-Analysis of Endoscopic Surveillance Studies for Detecting Dysplasia in Patients With Inflammatory Bowel Disease

**DOI:** 10.7759/cureus.58005

**Published:** 2024-04-10

**Authors:** Ghazala S Virk, Essam Rashad, Raheel Chaudhry, Mustafa M Moazam, Mohamed Mahbub, Aarish F Hanif, Yonas Tamene, Lydia Tadesse

**Affiliations:** 1 Internal Medicine, Avalon University School of Medicine, Ohio, USA; 2 Hospital Medicine, Parkview Regional Medical Center, Fort Wayne, USA; 3 Medicine, Baylor College of Medicine, Houston, USA; 4 Psychiatry, Texas Tech University Health Sciences Center El Paso, El Paso, USA; 5 Cardiovascular Medicine, Ain Shams University, Cairo, EGY; 6 Osteopathic Medicine, Arkansas College of Osteopathic Medicine, Fort Smith, USA; 7 Internal Medicine, California Institute of Behavioral Neurosciences & Psychology, Fairfield, USA; 8 School of Medicine, Addis Ababa University, Addis Ababa, ETH

**Keywords:** crohn’s disease, ulcerative colitis, endoscopy, surveillance, dysplasia, colon cancer surveillance, inflammatory bowel disease

## Abstract

Inflammatory bowel disease (IBD)is an extremely common gastrointestinal disorder that can give rise to dysplasia and colorectal cancer (CRC). There are various diagnostic methods but endoscopy has proved to be the best in the diagnosis, monitoring, and treatment of IBD. The objective of this review is to evaluate the efficacy of endoscopy in detecting patients with IBD. A structured search strategy on PubMed, Science Direct, and Google Scholar was used, as well as formal inclusion or exclusion, data extraction, validity assessment, and meta-analysis. RevMan 5.4 (Review Manager (RevMan) (Computer program). Version 5.4. The Cochrane Collaboration, 2020) was used for the meta-analysis, and forest plots were generated for each outcome separately. All of these studies are prospective cohorts and 11 of these are randomized controlled trials (RCTs). In IBD, both chromoendoscopy and white light endoscopy are useful in detecting dysplasia and neoplastic lesions. Furthermore, narrow-band imaging is a less time-consuming option for endoscopic surveillance. The meta-analysis also showed that chromoendoscopy is superior to other methods.

## Introduction and background

Gastrointestinal disorders are very common in the present population and inflammatory bowel disease (IBD) is one of them. Many people are affected by it every year [1]. Previously, many methods were used for the diagnosis but with the advent of science and technology, endoscopic techniques are widely in use. It is also an effective way of treatment monitoring because multiple treatment and management modalities can be evaluated by endoscopy [2,3]. To reflect the term mucosal examination and their walls, endoscopic techniques have proved to be the best. This is the gold standard and helps in determining the type and severity of IBD. This characterization helps in deciding the treatment plan for the disease [4,5]. Endoscopy, in conjunction with imaging, can also contribute to achieving the suspension of the disease. It is done by endoscopic measures known as mucosal healing (MH) or endoscopic remission (ER). By doing this, a focal area is targeted. It produces positive, long-lasting results [6,7]. Numerous meta-analyses have verified the remarkable results following MH treatment [8,9]. In addition to imaging and treatment, it can be used to prevent colorectal cancer (CRC) and dysplasia. The gold standard for endoscopic dysplasia detection in inflammatory bowel disease (IBD) is chromoendoscopy (CE). In CE, specific dyes are injected to enhance the visibility of the intestinal mucosal [10,11]. Different endoscopic tools are being used for the evaluation of disease [12]. Different methods are also being used to evaluate the staging of disease [12-15]. The purpose of this systematic review and meta-analysis is to assess how well endoscopic techniques and surveillance work for dysplasia detection.

## Review

Methodology

Search Strategy

All studies with endoscopic surveillance in dysplasia were searched. Different studies from peer-reviewed articles were studied and different qualitative and quantitative analyses were measured. All the searches were performed according to Preferred Reporting Items for Systematic Reviews and Meta-Analyses (PRISMA) guidelines. Study selection and data extraction were also done in accordance with PRISMA and PICO (Population, Intervention, Comparison, Outcome) criteria (Table 1).

**Table 1:** PICO table of the systematic review

Population (P)	Patients diagnosed with inflammatory bowel disease (IBD)
Intervention (I)	Endoscopic surveillance studies
Comparison (C)	Various methods or protocols employed in endoscopic surveillance
Outcome (O)	Detection of dysplasia in patients with inflammatory bowel disease

Eligibility Criteria

The review focuses on selecting studies that are very critical for understanding the endoscopic surveillance in patients with IBD having more emphasis on ulcerative colitis and Crohn's disease and the use of various techniques and guidelines for identification and management of dyslapsia. We prioritize research published in peer-reviewed journals and available in full text, ensuring the highest standard of evidence is included in our analysis. Conversely, we exclude studies that do not directly contribute to the objective of our review, including those not peer-reviewed, lacking in data, published in languages other than English, or involving child participants exclusively.

For a detailed breakdown of the inclusion and exclusion criteria employed in our review, see Table 2.

**Table 2:** Inclusion and exclusion criteria for the review

Inclusion Criteria	Exclusion Criteria
The study included patients with inflammatory bowel disease (IBD), including ulcerative colitis and Crohn's disease	Studies that are irrelevant
Endoscopic surveillance research that concentrated on identifying dysplasia	Types of publications that are not subjected to peer review are conference abstracts and posters
Endoscopic surveillance research employing different techniques or guidelines	Research with inadequate or missing data
Papers that are printed in journals with a peer review	Language publications (not just in English)
	Research with children as participants or information obtained exclusively from children

Information Sources

Several digital databases with relevant literature. PubMed, Google Scholar, and ScienceDirect are used.

Search Strategy

The research plan comprises a systematic and comprehensive search for pertinent literature regarding blog interventions' impact on mental health outcomes. Electronic databases, such as PubMed, Google Scholar, and Web of Science, are searched using a combination of keywords and MeSH terms. Boolean operators (AND, OR) are used to refine the strategy and add synonyms and variations of these terms to the search. To limit the results to human-subject studies published in peer-reviewed journals, search filters are used. In addition, references to pertinent articles and gray literature are manually searched in order to find possible studies that might have been overlooked by electronic searches. "Inflammatory bowel disease" OR "Crohn's disease" OR "Ulcerative colitis") AND "endoscopic surveillance" OR "colonoscopy" OR "endoscopy") AND "dysplasia detection" OR "meta-analysis" OR "systematic review") AND "clinical trial" OR "randomized controlled trial" was the search strategy that was employed.

Selection Process

The articles were screened twice. This review generated a list of potential papers. In the second stage of screening, the full text of the articles was used.

For each eligible paper, uniform data extraction tables were tabulated. Any disagreements were resolved through discussion with the final decision by the senior author. The meta-analyses were chosen because their outcome measures were similar. This meta-analysis excluded studies that did not have enough similar data or had poor methodological quality.

Data Collection

We searched peer-reviewed journals and publications for literature that met the inclusion criteria. To reduce the risk of publication bias, peer-reviewed journals were investigated after a thorough review of the literature. Based on the inclusion and exclusion criteria, we worked to "include" or "exclude" eligible studies. For the final review and analysis, 68 studies were considered. Studies that failed to meet the screening criteria were labeled "exclusion" or "dispute." Before excluding a study from the literature, exclusion reasons were presented. It was occasionally the result of a combination of several exclusionary factors.

Results

Data Items

**Figure 1:**
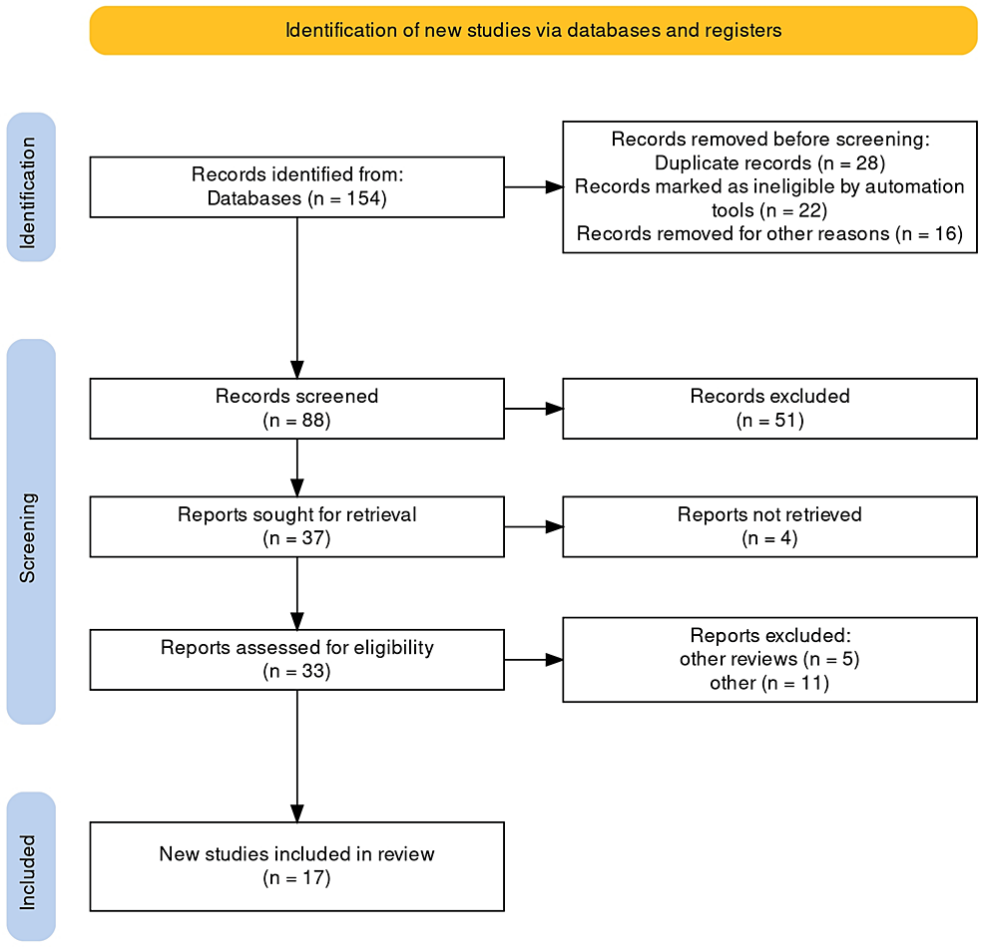
PRISMA flowchart of the studies included Articles were excluded based on eligibility criteria. Other systematic reviews and meta-analyses were not part of the review. Studies with irrelevant or wrong study outcomes were excluded. The predetermined eligibility criterion, which might have included elements like study designs, participants' criteria, intervention type, outcome measures, and publication date was used to exclude articles. Additional systematic reviews and meta-analyses were excluded from this review to keep the focus on original research studies. To ensure the accuracy and relevance of the results, studies with irrelevant or incorrect outcomes that did not align with the review's objective were also excluded. PRISMA: Preferred Reporting Items for Systematic Reviews and Meta-Analyses

Searching PubMed, Science Direct, and Google Scholar yielded all articles evaluating endoscopic surveillance for dysplasia detection. For statistical analysis, RevMan 5.4 software (Review Manager (RevMan) (Computer program). Version 5.4. The Cochrane Collaboration, 2020) was used. One hundred fifty-four articles were extracted after extensive research across all databases. The number of studies was reduced to 88 after duplicates were removed. This screening includes removing review articles as well as articles unrelated to the study. Seventeen studies were included in the systematic review and meta-analysis (Figure 1).

Study Characteristics

The final sample for the systematic analysis included 17 peer-reviewed studies. Table 3 contains a summary of the characteristics and demographic information of the included trials.

**Table 3:** Summary of all the included studies Sources: [16-32] IBD: Inflammatory Bowel Disease; CRC: Colorectal Cancer; CE: Chromoendoscopy; WLE: White Light Endoscopy; FVC: Forward-Viewing Colonoscopy; FUSE: Full-Spectrum Endoscopy; CGE: Chromoscopy-Guided Endoscopy; ETI: Endoscopic Trimodal Imaging; CE: Chromoendoscopy; NBI: Narrow-Band Imaging; UC: Ulcerative Colitis; AFI: Acute Fibrile Illness

Study	Study Design	Country	Sample Size	Type	Outcome Measures	Results
Leong et al. 2017 [16]	Prospective crossover study	Australia	Crohn’s disease=23, ulcerative colitis=29	Forward-viewing colonoscopy (FVC) and full spectrum endoscopy (FUSE)	Dysplastic lesions	When compared to conventional forward-viewing colonoscopy, panorama views obtained by full-spectrum endoscopy were found to increase the number of dysplastic lesions detected in IBD patients undergoing surveillance colonoscopy.
Jimeno et al. 2020 [17]	Prospective cohort study	Spain	176	Endoscopic biopsies	NA	A large number of samples must be examined in order to improve consistency in the diagnosis and grading of IBD-related dysplasia.
Alexandersson et al. 2017 [18]	Randomized controlled trial	Sweden	305	High-definition chromoendoscopy and white-light endoscopy	NA	In terms of dysplasia detection, HD-CE outperforms HD-WLE.
Freire et al. 2014 [19]	Randomized trial	Portugal	145	Chromoendoscopy-guided endomicroscopy and conventional colonoscopy	White light endoscopy vs chromoendoscopy	When screened endoscopically, CGE does not improve the detection of IN in patients with longstanding UC who do not have primary sclerosing cholangitis or a history of IN. CGE takes longer than CC, but it reduces the number of biopsies performed and significantly increases the IN per biopsy yield. Endomicroscopy is a trustworthy method of detecting IN.
Yang et al. 2019 [20]	Prospective randomized controlled trial	Korea	HDWL-R group (n 5 102) or HDCE-T group (n 5 108)	High-definition chromoendoscopy versus high-definition white light colonoscopy	Dysplastic lesions vs white light endoscopy vs chromoendoscopy	HDCE-T did not outperform HDWL-R in detecting dysplasia.
Vleugels et al. 2018 [21]	Multicenter randomized controlled trial	Netherlands	210	Endoscopic trimodal imaging (ETMI) and chromoendoscopy (CE)	NA	Endoscopic differentiation of dysplastic lesions detected during long-term UC surveillance using ETMI and CE appears to have limited sensitivity.
Iacopini et al. 2015 [22]	Prospective case series	Italy and Japan	9 patients	Curative endoscopic submucosal dissection	NA	In IBD patients, ESD achieves curative resections, but the procedure is difficult due to the high prevalence of submucosal fibrosis.
Iacucci et al. 2018 [23]	Randomized trial	Canada	270	High-definition colonoscopy alone, with high-definition dye spraying and electronic virtual chromoendoscopy	White light endoscopy vs chromoendoscopy	VCE or HD-WLE were equally good as compared to chromoendoscopy.
Kandiah et al. 2020 [24]	Multicentre randomized controlled trial	United Kingdom	188	Virtual chromoendoscopy	White light endoscopy vs chromoendoscopy	The detection of neoplasia by HDV and HDWL was not significantly different. During a targeted biopsy or resection, almost all neoplasia was discovered. Non-targeted biopsies do not provide any additional benefit.
Bisschops et al. 2017 [25]	Prospective randomized controlled trial	Belgium	131	Chromoendoscopy (CE) and narrow-band imaging (NBI)	NA	CE and NBI do not detect colitis-associated neoplasia in the same way. NBI may eventually replace traditional CE due to its longer withdrawal time and ease of use.
van den Broek et al. 2008 [26]	Randomized comparative trial of tandem colonoscopies	Netherlands	50	High-resolution endoscopy and autofluorescence imaging	NA	Autofluorescence imaging improves neoplasia detection in ulcerative colitis patients while reducing the yield of random biopsies. The pit pattern NBI analysis predicts histology with moderate accuracy, whereas AFI color appears useful in ruling out the presence of neoplasia.
van den Broek et al. 2010 [27]	Randomized crossover trial	Netherlands	48	High-definition endoscopy versus narrow-band imaging	NA	NBI is ineffective at distinguishing neoplastic from nonneoplastic mucosa.
Pellisé et al. 2011 [28]	Prospective, randomized, crossover study	Spain	80	Narrow-band imaging and chromoendoscopy	NA	NBI cannot be recommended as a standard technique due to the high rates of NBI lesions and patient misses.
Kiesslich et al. 2007 [29]	RCT	Germany	161	Chromoscopy-guided endomicroscopy	White light endoscopy vs chromoendoscopy	Endomicroscopy with in vivo histology can determine whether UC lesions identified by chromoscopy should be biopsied, increasing diagnostic yield, and reducing the number of biopsy examinations required.
Picco et al. 2013 [30]	RCT	USA	75	Chromoendoscopy	White light endoscopy vs chromoendoscopy	Indigo carmine CE produced high levels of interobserver agreement for polyp detection, acceptable withdrawal times, and improved dysplasia detection in UC surveillance. These findings are encouraging for the implementation of chronic UC CE programs.
Mohammed et al. 2015 [31]	RCT	USA	103	High-definition white light endoscopy (HDWLE) vs. high-definition chromoendoscopy (HDCE)	White light endoscopy vs chromoendoscopy	HDCE improves the detection of dysplastic lesions during surveillance endoscopy in patients with long-standing UC and should be used in these patients.
Park et al. 2006 [32]	RCT	NA	210	White light endoscopy (WLE) versus high definition with chromoendoscopy (HDCE	White light endoscopy vs chromoendoscopy	CE was better than WLE in detection but not less effective.

Meta-Analysis

This study gathered data from various research studies. Mean, standard deviations, events, and total were extracted for each variable. When these specific data were unavailable, the means and standard deviations of the intervention and control groups' outcome measurements were used as a substitute. Every trial with relevant outcome data was included, and primary analyses were performed for each score. RevMan 5.4 from the Cochrane database was used.

Dysplastic Lesions Identified

**Figure 2:**
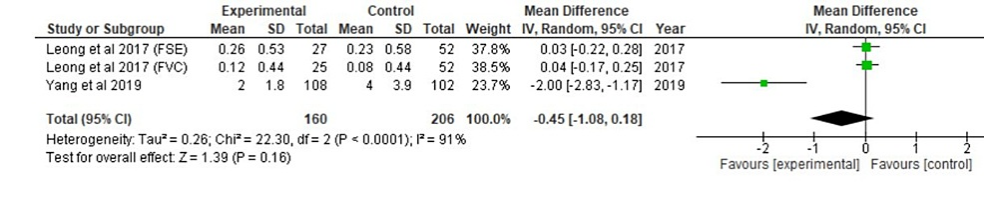
Forest plot of the identified dysplastic lesions Sources: [16,20] FVC: Forward-Viewing Colonoscopy, FSE: Full-Spectrum Endoscopy, CI: Confidence Interval, SD: Standard Deviation

Using continuous data, the forest plot was created for three different studies. The overall effect size was calculated using Cohen's d as d=-0.45 and CI=95% (-1.08, 0.18). The calculated heterogeneity is as follows: I2=91%, Chi^2^=22.30, df=2 (p-value <0.0001). The overall effect analysis, Z=1.39 (p=0.16), was discovered (Figure 2).

White Light Endoscopy vs Chromoendoscopy

**Figure 3:**
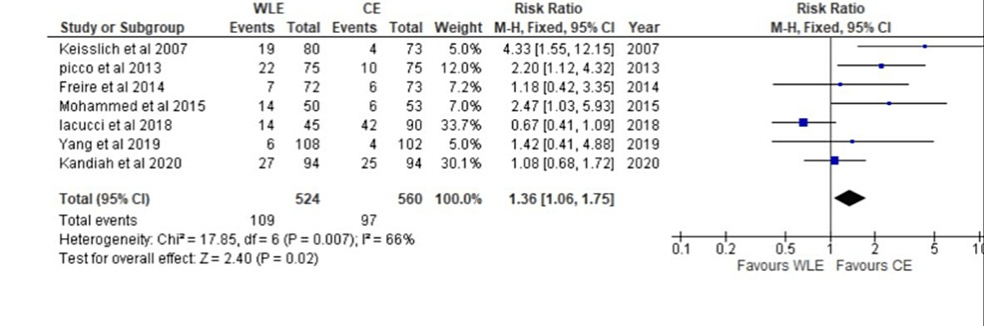
Forest plot of comparison of white light endoscopy (WLE) and chromoendoscopy (CE) Sources: [19,20,23,24,29,30,31] CE: Chromoendoscopy, WLE: White Light Endoscopy, CI: Confidence Interval

A Forest plot was created for each of the seven different studies to collect dichotomous data. A fixed-effects model was used with the Mantel-Haenszel test. The horizontal axis was used to calculate the CI (CI=95%), and the plot's "point estimation" was represented by green squares. The vertical line in the center indicates "no effect." The result was d=1.36, CI=95% (1.06, 1.75). Chi^2^=17.85, df=6 (p-value=0.007), and I2=66% were the calculated heterogeneities. The overall effect was determined to be Z=2.40 (p=0.02) after analysis. Individual effects in each study favored the chromoendoscopy group (Figure 3).

To evaluate the overall effectiveness of endoscopic surveillance in identifying dysplasia in patients with IBD, our study combined data from several studies. When compared to non-surveillance or less intensive surveillance approaches, we discovered that endoscopic surveillance was linked to a significantly higher detection rate of dysplasia. These results support earlier studies that have suggested the importance of routine endoscopic monitoring in the treatment of IBD patients, especially in detecting dysplasia early on when it may be curable. According to the findings of the systematic review, endoscopy is superior in detecting dysplasia in patients with IBD. In the context of IBD, both chromoendoscopy and white light endoscopy have been shown to be effective in detecting dysplasia and neoplastic lesions. Furthermore, the incorporation of narrow-band imaging in endoscopic surveillance provides an additional, albeit less time-consuming option. However, it is noted that its effectiveness is lower than that of other endoscopic methods. Our meta-analysis's findings have significant clinical ramifications for how IBD patients are treated. Through endoscopic surveillance, dysplasia can be identified early and treated promptly, potentially improving patient outcomes and lowering mortality. Furthermore, our results corroborate the current guidelines for routine endoscopic surveillance in high-risk groups, including individuals with a history of dysplasia or long-standing, extensive colonic disease. This comprehensive analysis contributes to the optimization of clinical practices and future research in this domain by providing valuable insights into the comparative efficacy and efficiency of different endoscopic approaches for dysplasia detection in IBD. Compared to CE, WLE detects fewer non-polypoid dysplastic lesions. The use of CE in patients with IBD is required in clinical trials, and it is preferable to add the magnification technique to improve the detection rate [33]. According to another review, RCTs compared CE to HD-WLE rather than HD-WLE to CE. Dedicated studies to compare the accuracy of CE versus high-definition endoscopy will almost certainly be conducted shortly. Importantly, the noninferiority of HD-WLE to CE has already been reported [34]. Another review by Gondal et al. found that high-definition (HD) imaging was superior to detecting dysplasia in ulcerative colitis (UC) patients regardless of the image-enhancing modality used. Notably, current colorectal cancer surveillance guidelines for UC patients show that HD consistently outperforms other options for dysplasia detection. The study emphasizes the uncertainty about the best modality for surveillance colonoscopies in UC, but it strongly advocates for the use of HD, claiming its superiority over white light standard definition, which was identified as the least effective option. The study recommends increased dysplasia detection rates and targeted biopsies, highlighting the potential for improved dysplasia detection rates and targeted biopsies [35]. The most popular method for visually evaluating colon lesions is white light endoscopy; however, it is not as good at improving the visualization of surface and vessel patterns. Also known as chromoendoscopy, the colon is sprayed with chemical dyes to achieve greater contrast outcomes compared to WL imaging [36].

All the 17 studies included in this systematic review and meta-analysis are prospective and 11 are RCTs. The limitations noted in this review are: variability in study designs, such as differences in patient populations and surveillance protocols, introduces heterogeneity in the results synthesis. Concerns have been raised about the representation of studies with positive or statistically significant findings due to the possibility of publication bias. The incompatibility of data reporting, variations in endoscopic techniques, and variations in dysplasia definitions among studies exacerbate the challenge of comparing and generalizing results. Future investigations in this field ought to focus on resolving the constraints noted in our analysis and providing more clarification on the best practices for endoscopic monitoring of IBD patients. Larger sample sizes and standardized surveillance protocols in longitudinal studies are required to confirm our results and offer stronger evidence for clinical decision-making. Furthermore, the creation and assessment of innovative endoscopic methods, like chromoendoscopy and sophisticated imaging modalities, may improve dysplasia detection.

## Conclusions

Ultimately, the findings demonstrate that dysplasias in the setting of inflammatory bowel disease (IBD) can be found by endoscopic surveillance. Chromoendoscopy is superior to narrow-band imaging and white light endoscopy in the detection of dysplasia. The evidence suggests that the precision and accuracy of dysplasia identification in IBD patients can be enhanced by the use of chromoendoscopy during endoscopic surveillance. By highlighting the significance of using the right endoscopic techniques to identify dysplasia and, ultimately, improve patient outcomes in the treatment of inflammatory bowel disease, these findings add to clinical practice.
